# Effects of different recovery strategies following a half-marathon on fatigue markers in recreational runners

**DOI:** 10.1371/journal.pone.0207313

**Published:** 2018-11-09

**Authors:** Thimo Wiewelhove, Christoph Schneider, Alexander Döweling, Florian Hanakam, Christian Rasche, Tim Meyer, Michael Kellmann, Mark Pfeiffer, Alexander Ferrauti

**Affiliations:** 1 Faculty of Sport Science, Ruhr-University Bochum, Bochum, Germany; 2 Institute of Sports Science, Johannes-Gutenberg University, Mainz, Germany; 3 Institute of Sports and Preventive Medicine, Saarland University, Campus, Saarbrücken, Germany; 4 Faculty of Health and Behavioural Sciences, The University of Queensland, Campbell Road St Lucia Campus, QLD, Brisbane, Australia; University of Essex, UNITED KINGDOM

## Abstract

**Purpose:**

To investigate the effects of different recovery strategies on fatigue markers following a prolonged running exercise.

**Methods:**

46 recreational male runners completed a half-marathon, followed by active recovery (ACT), cold water immersion (CWI), massage (MAS) or passive recovery (PAS). Countermovement jump height, muscle soreness and perceived recovery and stress were measured 24h before the half-marathon (pre), immediately after the recovery intervention (post_rec_) and 24h after the race (post_24_). In addition, muscle contractile properties and blood markers of fatigue were determined at pre and post_24_.

**Results:**

Magnitude-based inferences revealed substantial differences in the changes between the groups. At post_rec_, ACT was harmful to perceived recovery (ACT vs. PAS: effect size [ES] = −1.81) and serum concentration of creatine kinase (ACT vs. PAS: ES = 0.42), with CWI being harmful to jump performance (CWI vs. PAS: ES = −0.98). It was also beneficial for reducing muscle soreness (CWI vs. PAS: ES = −0.88) and improving perceived stress (CWI vs. PAS: ES = −0.64), with MAS being beneficial for reducing muscle soreness (MAS vs. PAS: ES = −0.52) and improving perceived recovery (MAS vs. PAS: ES = 1.00). At post_24_, both CWI and MAS were still beneficial for reducing muscle soreness (CWI vs. PAS: ES = 1.49; MAS vs. PAS: ES = 1.12), with ACT being harmful to perceived recovery (ACT vs. PAS: ES = −0.68), serum concentration of creatine kinase (ACT vs. PAS: ES = 0.84) and free-testosterone (ACT vs. PAS: ES = −0.91).

**Conclusions:**

In recreational runners, a half-marathon results in fatigue symptoms lasting at least 24h. To restore subjective fatigue measures, the authors recommend CWI and MAS, as these recovery strategies are more effective than PAS, with ACT being even disadvantageous. However, runners must be aware that neither the use of ACT nor CWI or MAS had any beneficial effect on objective fatigue markers.

## Introduction

A long-distance running exercise such as a half-marathon or a marathon is one of the most strenuous activities because it combines a prolonged duration with an eccentric component and a relatively high exercise intensity [1]. Although the resultant post-exercise fatigue is based on many factors (e.g. related to the central nervous system and/or peripheral factors within the skeletal muscles) that have not yet been fully explored because of their complexity, it is usually reflected in a substantial decrease in muscle performance that can last up to several days [2, 3]. Thus, the use of recovery strategies seems to be required to overcome the demands of, for example, a half-marathon- or a marathon-type exertion, to increase the athlete’s well-being and allow them to return to normal training as quickly as possible without increased risk of injury or illness [4, 5].

A variety of recovery methods can help to alleviate the deleterious effects of muscle fatigue caused by strenuous exercise [6]. Apart from nutritional strategies, three of the best known recovery interventions are active recovery (also referred to as active cool-down and usually consisting of aerobic-type whole-body activities, such as running, biking or swimming) [7], cold water immersion [8] and massage [9]. Although numerous studies have investigated their efficacy, most have used non-functional exercises to induce fatigue. Unfortunately, there are limited studies investigating the potential effectiveness of recovery strategies in road running. However, post-exercise fatigue varies in type and degree depending on the characteristics of the exercise that brings on the fatigue [2]. Therefore, the findings of the majority of these studies may not apply to the actual practice of long-distance runners.

Only one previous study investigated the effect of cold water immersion on the markers of recovery following a marathon. Wilson et al. [10] showed that cold water immersion is no more effective than a placebo effect in improving functional recovery or perceptions of muscle soreness and exercise stress following a road race. Similarly, Dawson et al. [11] observed no effect of massage applied after a half-marathon road race on the recovery of muscles, either in terms of physiological measures of strength and swelling or perceived muscle pain. Sherman et al. [12] compared the effect of running at low intensities (i.e. active recovery) with that of passive recovery over one week following a marathon. Their findings showed that active recovery did not facilitate the restoration of marathon-induced reductions in muscle function.

In summary, although the incorporation of recovery strategies into a period of regeneration is becoming increasingly popular across both recreational and elite level runners, the aforementioned studies unanimously report that recovery interventions do not promote recovery from marathon-induced fatigue. However, because of the small number of studies that have examined the use of recovery methods in long-distance running, little evidence seems to support the efficacy or inefficacy of active recovery, cold water immersion, or massage in recovery following a marathon-type exertion. Moreover, to our knowledge, no study has yet compared the effects of various recovery strategies following road running within a single-study setting. Therefore, this study aimed to compare the effects of active recovery, cold water immersion, massage and passive recovery on fatigue markers in recreational runners following the completion of a half-marathon run.

## Materials and methods

### Participants

A total of 46 healthy and male recreational runners were recruited from the participants of an official competitive half-marathon race to take part in this study. The baseline physical and performance characteristics of the 46 runners are shown in Table 1. After being informed about the exercise protocol and all the possible risks associated with participating in the investigation, the runners provided their written consent to participate in all procedures. To ensure that there were no contraindications to vigorous exercise, all the subjects completed the Physical Activity Readiness Questionnaire before any testing took place. The research was approved by the ethics committee of the Medical Faculty of the Ruhr-University Bochum and conducted according to the guidelines of the Declaration of Helsinki.

**Table 1:** Physical and performance characteristics of the runners.

	Recovery groups														
	PAS (n = 12)			ACT (n = 13)			CWI (n = 11)			MAS (n = 10)			Overall (n = 46)		
Age (years)	31.3	±	12.3	30.2	±	8.6	31.5	±	10.2	28.9	±	12.2	30.5	±	10.9
Weight (kg)	74.8	±	7.6	74.9	±	8.0	78.3	±	8.3	73.9	±	4.9	75.5	±	7.5
Height (cm)	180.8	±	4.1	177.9	±	5.6	181.1	±	7.7	178.1	±	6.0	179.4	±	6.2
BMI (kg/m^2^)	22.9	±	2.6	23.6	±	2.2	23.9	±	2.8	23.4	±	2.3	23.5	±	2.4
AT (m/s)	3.57	±	0.48	3.63	±	0.38	3.65	±	0.31	3.61	±	0.21	3.62	±	0.36
Finish time (min)	116	±	25	107	±	12	111	±	10	116	±	13	112	±	16
%AT	92.1	±	6.2	92.9	±	5.3	89.0	±	5.8	86.2	±	9.4	90.2	±	7.4
RPE (0–10)	7.5	±	1.9	7.1	±	1.3	6.7	±	1.7	7.4	±	1.7	7.2	±	1.7

Data are shown as the mean ± SD; no significant differences (*p* > 0.05) in the mean values among the recovery groups; PAS, passive recovery; ACT, active recovery; CWI, cold water immersion; MAS, massage; BMI, body mass index; AT, anaerobic threshold; %AT, mean percentage running velocity during the half-marathon associated with AT; RPE, rating of perceived exertion.

### Experimental design

A matched quartet controlled parallel design was used to investigate the effect of three different recovery strategies on fatigue markers following a half-marathon. The runners participated in an official competitive half-marathon race. Within 15 min after the event, the subjects undertook one of four recovery protocols: active recovery (ACT), cold water immersion (CWI), massage (MAS), or passive recovery (PAS). For the assignment to ACT, CWI, MAS, or PAS, the runners were matched into homogeneous quartets according to age and anaerobic lactate threshold (AT). Then, within each quartet, the subjects were randomly assigned to one of the four experimental groups (Table 1). The runners’ AT was determined in an incremental field test conducted one week prior to the race [13].

Countermovement jump (CMJ) height, muscle soreness and perceived recovery and stress were then measured 24 h before the half-marathon (pre), immediately after the recovery intervention (post_rec_) and 24 h after the race (post_24_). In addition, the muscle contractile properties and the serum concentrations of creatine kinase (CK), c-reactive protein (CRP), urea, free-testosterone (f-T) and insulin-like growth factor 1 (IGF-1) were determined 24 h before and after the half-marathon. These markers were selected based on preliminary research by our working group, which investigated the sensitivity of different markers for assessment of fatigue and recovery [14−16]. Furthermore, the participants were asked to maintain their normal dietary intake and to avoid consuming any nutritional supplements, caffeine, or alcohol during the experimental period (i.e. one week before the competition until the end of the last testing session). In this regard, the subjects were verbally questioned several times during the experimental period to ensure that they had adhered to the dietary rules. The verbal feedback from the runners suggests that compliance with nutritional rules was good. In addition, for the first two hours after the race, all participants were provided with the same food and drinks (i.e. bananas, cereal bars and water without additives).

### Procedures and measurements

#### Incremental field test

To determine the AT, a progressive incremental exercise test was conducted on a 400 m outdoor running track (tartan surface). For this test, which was modified according to Mader et al. [13], the initial running speed was set to 2.0 m·s^-1^ and increased by 0.4 m·s^-1^ after each velocity level. Cones were set at 50 m intervals along the 400 m track (inside the first lane). The running pace was dictated by audio signals, and the participants had to be within 2 m of the cones at each signal. The test was terminated when the subjects were behind a cone for three consecutive times. During the first velocity level (i.e. 2.0 m·s^-1^), the participants had to run 400 m. The subjects had to run 800 m to complete the subsequent three stages (i.e. 2.4, 2.8 and 3.2 m·s^-1^). During levels 3.6, 4.0, 4.4, 4.8 and 5.2 m·s^-1^, the runners had to run 1,200 m. Only one runner reached the end of level 5.2 m·s^-1^.

Capillary blood samples were taken from a hyperemised earlobe before the test, during a 60 s break immediately following each submaximal velocity level, and at the time point of exhaustion. These samples were analysed for blood lactate concentration. The blood samples were taken with 20 μL capillaries, haemolysed in 1 mL microtest tubes and analysed enzymatic amperometrically by the Biosen S-Line Sport (EKF-diagnostic GmbH, Barleben, Germany). From the resultant La values, the AT was determined according to Heck et al. [17] and Mader et al. [13].

#### Jump performance

CMJs were preceded by a short standardized warm-up and performed on a contact platform (Haynl-Elektronik GmbH, Schönebeck, Germany), with the hands placed on the hips. In performing the CMJs, the runners dropped down to a self-selected level before jumping to the maximum height. Flight time was used to calculate jump height. Three CMJs were performed with approximately 10 s of passive recovery between efforts. The best CMJ value over the three attempts was then computed [18]. Previously measured reliability scores for the CMJ test were regarded as highly reliable (unpublished results, 2013: n = 38, CMJ [cm]: ICC = 0.92, TE = 1.86, CV = 3.7%).

#### Muscle contractile markers

To assess the contractile characteristics of the rectus femoris muscle of the dominant lower limb, Tensiomyography (TMG) was used under laboratory conditions. This technique causes the radial displacement of the muscle belly in response to a submaximal (i.e. below the voluntary maximal activation) electrical stimulus triggered by a specific electrical stimulator (TMG-S2) that is conducted through the underlying muscle tissue. These displacements were then recorded on the skin surface using a displacement sensor tip with a spring constant of 0.17 N∙mm^-1^ along with the TMG-OK 3.0 software (TMG-BMC, Ljubljana, Slovenia).

The sensor was positioned perpendicular to the thickest part of the muscle belly, which was established visually and through palpation during a voluntary contraction, and the self-adhesive electrodes were placed symmetrically approximately 5 cm away from the sensor. Once the exact position for the sensor and electrodes was established, it was marked with a dermatological pen and kept constant during the experimental period. The individual maximal electrical stimulation and muscle belly displacement were found by progressively increasing the electric current by 20 mA until no further displacement of the muscle belly could be produced. Each stimulation was separated by 10 s intervals to minimise the effects of fatigue and potentiation. The average values from the two maximal twitches were used for further analysis. The rectus femoris muscle was assessed in the supine position, and an internal knee angle of 120° was maintained using supporting pads [15, 16].

The TMG measures included Dm and Tc. Dm is equivalent to the maximal radial deformation of the muscle belly, which is representative of muscle tone and contractile force. Tc is the deformation time between 10% and 90% Dm and refers to the time of the muscle radial deformation, which in turn indicates the time and speed of force generation [15]. Dm and Tc were the main parameters in this trial because of sufficient reliability scores (unpublished results, 2013: n = 20, Dm [mm]: ICC = 0.87, TE = 0.97, coefficient of variation (CV) = 12.9%; Tc [ms]: ICC = 0.90, TE = 1.86, CV = 5.8%).

#### Subjective markers

Muscle soreness was recorded using a visual analogue scale (VAS). The VAS consisted of a 100 mm line, the endpoints of which were labelled as ‘no pain’ (left) and ‘unbearable pain’ (right). The VAS, which has been shown to be valid and reliable in previous research, was selected because it is commonly used to detect delayed onset muscle soreness [19]. Subjects were asked to palpate their lower limb muscles and then draw a vertical line at a point on the scale that best represented their general amount of muscle pain at the time of the measurement. Their score was determined from the distance in mm from the left border of the scale to the point marked [19].

Perceived recovery and stress states were determined using the Short Recovery and Stress Scale (SRSS; [20]). The participants were requested to provide responses to eight items on a zero (does not apply at all) to six (fully applies) rating scale. Numbers one to five on this scale were undefined and used to delineate the degrees of perceived recovery and stress between the two ends of the scale. The items used in this study were ‘Overall Recovery’ and ‘Overall Stress’ [20].

#### Blood markers

Venous blood samples were collected 24 h before and after the half-marathon from an antecubital arm vein using a 20-gauge disposable Safety-Multifly needle (Sarstedt AG & Co., Nümbrecht, Germany) while the subject was in the supine position. Samples were collected into 7.5 mL serum gel tubes with a clotting activator (Sarstedt AG & Co., Nümbrecht, Germany) and subsequently centrifuged within 20 min after sampling. The resultant serum was separated from the other compounds, pipetted into micro tubes (Sarstedt AG & Co., Nümbrecht, Germany), frozen at −80°C within 60 min from blood collection and then stored for later analysis.

Automated techniques (UniCell DxC 600 Synchron; Beckmann Coulter GmbH, Krefeld, Germany) were used to analyse the serum concentration of CK, CRP and urea. f-T and IGF-1 were examined in one run using commercially available ELISA kits (f-T: LDN Labor Diagnostika Nord GmbH & Co. KG, Nordhorn, Germany; IGF-1: mediagnost GmbH, Reutlingen, Germany; all purchased via Beckmann Coulter GmbH, Krefeld, Germany). The respective protocols supplied by the manufacturer were strictly followed. Each sample was analysed in double, and the mean of the two measurements was used as the estimate of the sample concentration [14].

In addition, capillary blood samples were taken from the hyperemised earlobe immediately after the recovery intervention and analysed for the serum concentration of CK. The samples were collected with a 200 μL capillary blood collection system prepared for serum collection with a coagulation inducer and a separating gel (KABE Labortechnik GmbH, Nümbrecht-Elsenroth, Germany), positioned upright to clot at room temperature for 10 min, subsequently centrifuged and then analysed by the COBAS INTEGRA 400 plus (Roche Diagnostics, Basel, Switzerland).

### Competitive race

All of the runners participated in the same official competitive half-marathon race, and they climbed a total altitude of 450 m. The average time required for the race was between 107 min and 116 min in the four experimental groups, and it corresponded to a mean percentage running velocity associated with the AT of 86.2%–92.9%. The runners’ perception of the overall difficulty of the race, which was recorded immediately before performing the recovery interventions using a category ratio scale [21], ranged on average from 6.7 to 7.5 (i.e. very hard; Table 1). The external and internal loads were not significantly different (p > 0.05) among the recovery groups.

### Recovery interventions

Within 15 min of completing the half-marathon race, all the runners commenced their assigned recovery intervention. Before and immediately after the interventions, all runners were provided with bananas, cereal bars and water. During PAS, the participants remained seated at rest on a bench for 15 min. ACT consisted of sub-maximal jogging for 15 min at a running speed equivalent to 60% of AT [22]. CWI was also applied continuously for 15 min, during which the subjects remained seated in tubs while immersing their lower limbs, ensuring that the iliac crest was fully submerged in the water bath. The water temperature was maintained at 15 ± 1°C by adding crushed ice as needed and controlled by the use of liquid-in-glass thermometers. This protocol was adopted because a recent review demonstrated the presence of a dose–response relationship with CWI, indicating that a water temperature of 11°C– 15°C and an immersion time of 11–15 min could provide the best results [23]. The participants were also instructed to perform circular motions with their legs every 2 min to prevent the formation of a warmer boundary layer surrounding the skin [8, 24].

MAS consisted of classical sports massage techniques and included effleurage (or stroking), petrissage (or kneading) and friction (or rubbing) [25]. The 20 min massage protocol was split into 5 min massage routines for each leg in both the prone and supine positions (Table 2). The massage was initially started with the subject in the prone position on a massage table, beginning with the right leg and then the left leg. Thereafter, the athlete was placed in the supine position and the massage continued, beginning again with the right leg [26]. Sports scientists certified in sports massage performed the massage therapy. Under verbal instructions recorded on an audiotape, the therapists performed the massage protocol throughout. The masseurs were requested to keep the depth and rate of the massage as consistent as possible [27]. Sunflower oil was used to decrease friction between the hands of the masseur and the skin of the subject. To ensure that CWI and MAS were implemented within 15 minutes after the race, five water tubs were used and five sports scientists performed the massage therapy.

**Table 2:** Massage protocol.

Time (mm:ss)	Technique/major body region	
	*Prone position*	*Supine Position*
00:00–00:15	Effleurage: upper thigh/hamstrings	Effleurage: upper thigh/quadriceps
00:15–00:45	Petrissage: upper thigh/hamstrings	Petrissage: upper thigh/quadriceps
00:45–01:00	Effleurage: upper thigh/hamstrings	Effleurage: upper thigh/quadriceps
01:00–01:30	Friction: upper thigh/hamstrings	Friction: upper thigh/quadriceps
01:30–01:45	Effleurage: upper thigh to popliteal fossa	Effleurage: upper thigh to knee
01:45–02:15	Petrissage: popliteal fossa	Friction: knee
02:15–02:30	Effleurage: popliteal fossa to lower leg	Effleurage: knee to shin
02:30–03:00	Petrissage: lower leg/calf	Effleurage: shin
03:00–03:15	Effleurage: lower leg/calf	Effleurage: shin to foot
03:15–03:45	Friction: lower leg/calf	Friction: ankle
03:45–04:00	Effleurage: calf to Achilles tendon	Effleurage: ankle and foot
04:00–04:30	Friction: Achilles tendon	Friction: foot
04:30–05:00	Effleurage: upper thigh and lower leg	Effleurage: upper thigh and lower leg

### Statistical analysis

Our statistical analyses were conducted using Microsoft Excel (version 15.17, Microsoft Corp., Redmond, WA, USA) and IBM SPSS Statistics (version 23, IBM Corporation, Amonk, New York, USA). Data were expressed as means ± standard deviations (SD) and tested for normal distribution using the Shapiro–Wilk test. Muscle soreness, overall recovery, overall stress, CK, CRP, urea and f-T were log transformed to reduce bias due to non-uniformity of error.

The magnitude of the within-group changes between testing sessions and the magnitude of the differences in the changes between the recovery groups were assessed using the effect size. The effect sizes were calculated as standardized mean differences using the mean pretest SD adjusted for sample size [28]. The threshold values of 0.00–0.19, 0.20–0.59, 0.60–1.19, 1.20–1.99 and > 2.00 were considered trivial, small, moderate, large and very large, respectively [29]. To assess the probability that the magnitude of the differences in the changes among the recovery groups is practically meaningful, 90% confidence intervals for the between-groups differences in the changes were estimated using a published spreadsheet [30]. A substantial difference in the changes was accepted when there was more than 75% likelihood that the true value of the standardised mean difference was greater than the smallest worthwhile change (SWC) [5, 29]. The SWC was calculated as 0.2 multiplied by the adjusted mean pretest SD in the fatigue markers [28].

In addition, a repeated-measure analysis of variance with the factors recovery intervention and time was used to determine the differences in the fatigue markers between the recovery groups as well as between testing sessions. Statistical significance was set to *p* < 0.05.

## Results

**Fig 1:**
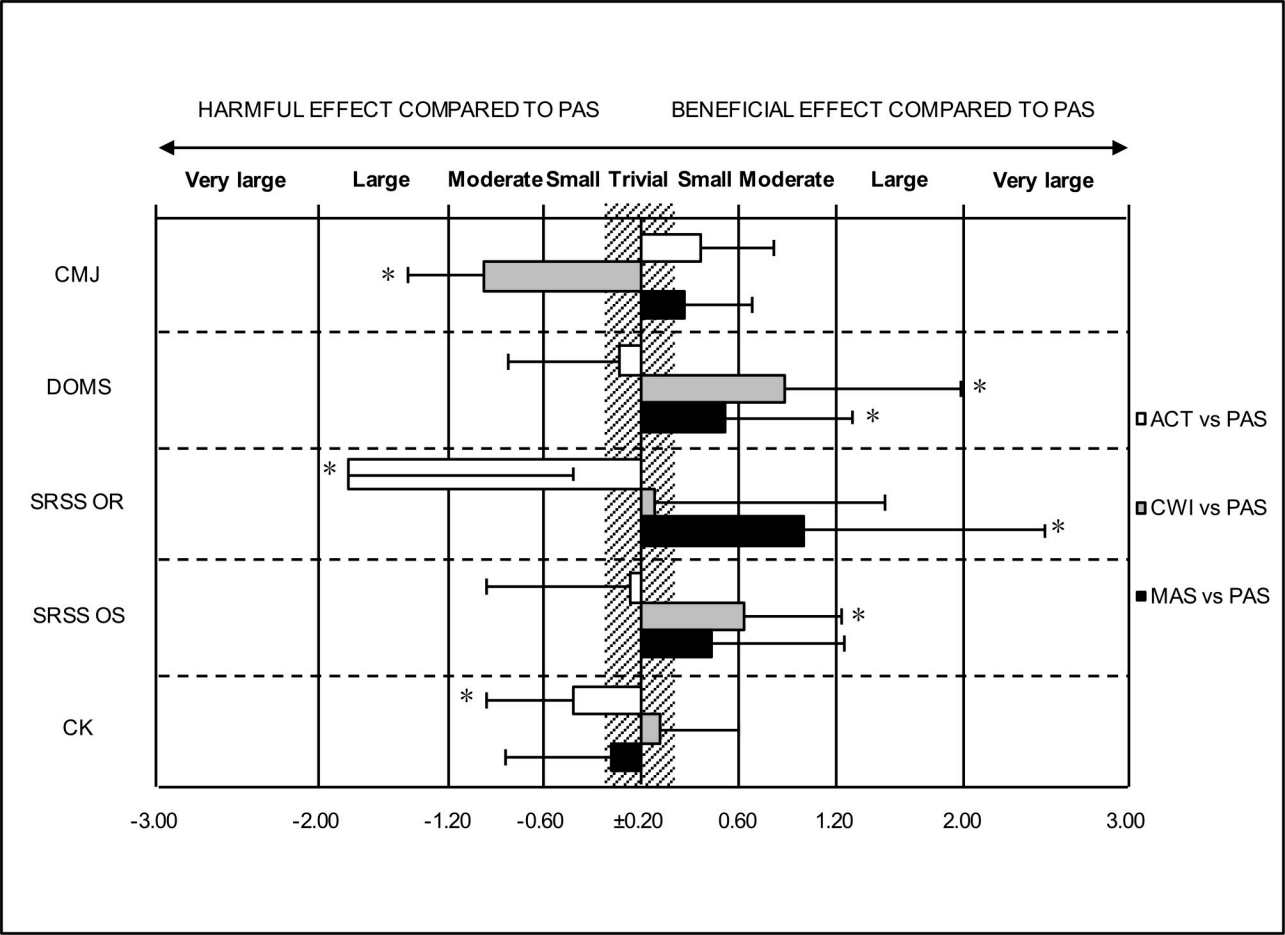
Standardised differences in the pre to post_rec_ changes (± 90% confidence intervals) between active recovery (ACT), cold water immersion (CWI), massage (MAS) and passive recovery (PAS) in the countermovement jump (CMJ) performance, perceived delayed onset muscle soreness (DOMS), perceived recovery (OR) and stress (OS) and the serum concentration of creatine kinase (CK). *75% likelihood that the true value of the standardised mean difference is greater than the smallest worthwhile change.

**Fig 2:**
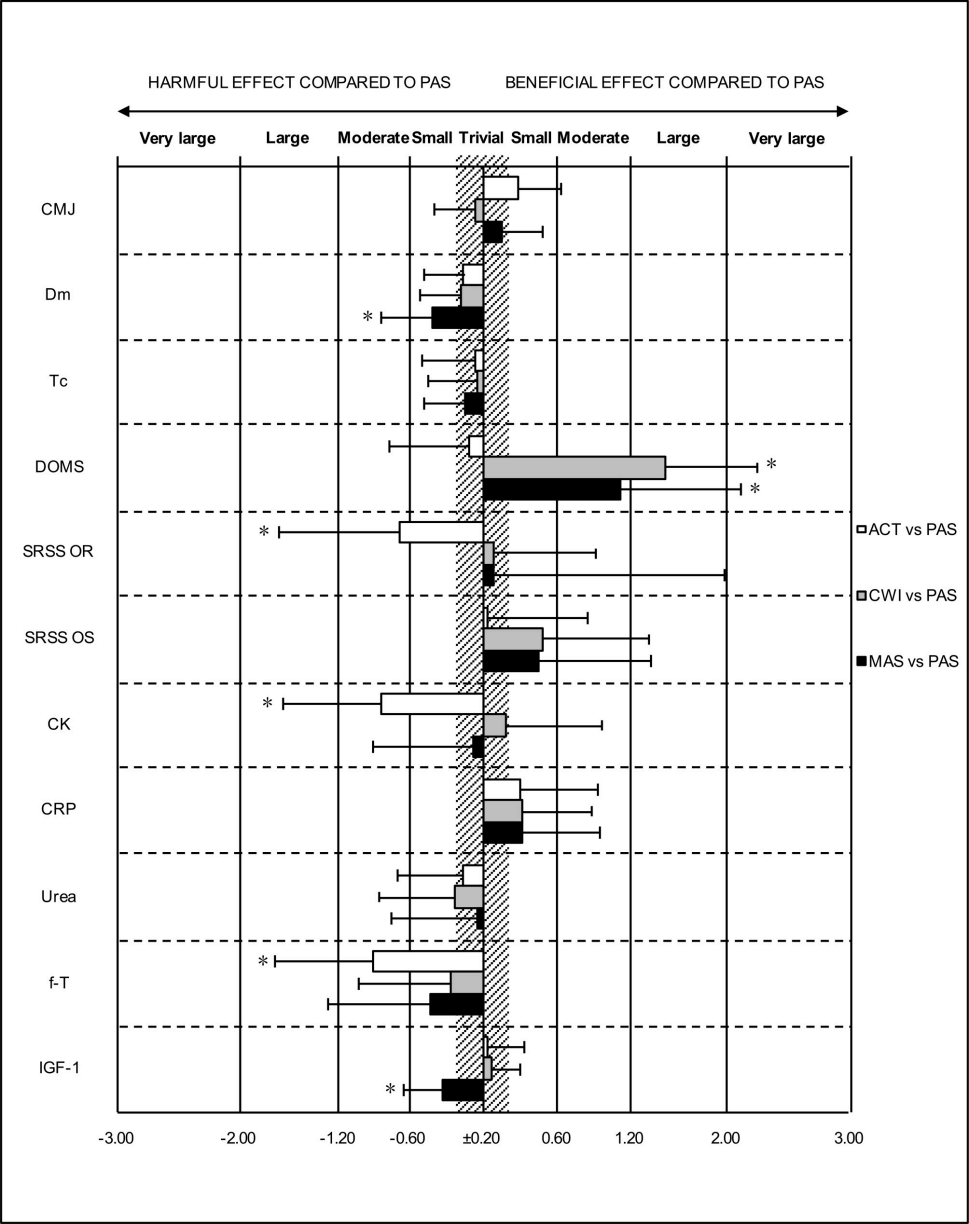
Standardised differences in the pre to post_24_ changes (± 90% confidence intervals) between active recovery (ACT), cold water immersion (CWI), massage (MAS) and passive recovery (PAS) in the countermovement jump (CMJ) performance, muscle contractile characteristics [i.e. muscle belly displacement (Dm) and contraction time (Tc)], perceived delayed onset muscle soreness (DOMS), perceived recovery (OR) and stress (OS) and the serum concentrations of creatine kinase (CK), c-reactive protein (CRP), urea, free-testosterone (f-T) and insulin like growth factor 1 (IGF-1) among the recovery groups. *75% likelihood that the true value of the standardised mean difference is greater than the smallest worthwhile change.

The mean (± SD) pre, post_rec_ and post_24_ values for the fatigue markers and the magnitudes of the within-group changes between testing sessions are presented in Tables 3 and 4. Magnitude-based inferences revealed substantial differences in the changes in the fatigue markers between the recovery groups (Figs 1 and 2).

**Table 3:** Countermovement jump performance, muscle contractile characteristics and perceived muscle soreness, recovery and stress 24 h before the half-marathon (pre), immediately after the recovery intervention (post_rec_) and 24 h after the half-marathon (post_24_).

Variable	Group	Pre			Post_rec_			Post_24_			Time	Intervention x time interaction
											*p*	*p*
CMJ (cm)	PAS	31.5	±	4.3	30.8	±	4.1^a^	30.6	±	4.8^a^	0.009	0.001
	ACT	31.2	±	5.3	32.3	±	6.4^b^	31.8	±	5.5^a^		
	CWI	33.6	±	5.4	27.9	±	3.8^c^	32.4	±	5.1^b^		
	MAS	32.9	±	5.4	33.5	±	5.3^a^	32.7	±	5.7^a^		
Dm (mm)	PAS	9.6	±	3.0				9.5	±	2.7^a^	0.020	0.314
	ACT	8.7	±	2.9				9.2	±	2.9^a^		
	CWI	8.0	±	1.9				8.5	±	1.6^a^		
	MAS	9.9	±	3.6				11.1	±	3.0^b^		
Tc (ms)	PAS	33.2	±	6.2				34.0	±	6.6^a^	0.010	0.938
	ACT	29.7	±	4.7				31.0	±	3.4^b^		
	CWI	29.7	±	5.1				30.8	±	5.2^b^		
	MAS	28.8	±	5.2				30.5	±	5.7^b^		
DOMS (mm)	PAS	0.6	±	0.5	4.6	±	2.3^e^	4.8	±	2.2^e^	0.001	0.016
	ACT	0.7	±	0.8	5.4	±	2.7^e^	5.4	±	2.6^e^		
	CWI	1.9	±	2.2	4.2	±	1.8^d^	3.8	±	2.8^c^		
	MAS	1.0	±	1.3	4.1	±	2.2^d^	3.3	±	2.9^d^		
SRSS OR (0–6)	PAS	4.0	±	1.7	2.8	±	1.5^d^	2.8	±	1.2^c^	0.001	0.022
	ACT	4.2	±	0.9	1.7	±	1.1^e^	2.7	±	1.2^d^		
	CWI	4.4	±	0.9	3.1	±	1.6^c^	2.9	±	0.9^c^		
	MAS	4.2	±	1.0	3.8	±	1.3^b^	3.6	±	1.8^c^		
SRSS OS (0–6)	PAS	1.6	±	1.1	4.1	±	1.6^d^	2.9	±	1.6^c^	0.001	0.219
	ACT	1.6	±	1.4	4.4	±	1.1^d^	2.9	±	1.4^c^		
	CWI	2.2	±	1.3	3.1	±	1.6^b^	3.2	±	1.5^b^		
	MAS	1.7	±	1.1	3.3	±	1.5^c^	2.7	±	1.9^b^		

Data are shown as the mean ± SD

a, trivial change (ES = 0.00–0.19)

b, small change (ES = 0.20–0.59)

c, moderate change (ES = 0.60–1.19)

d, large change (1.20–1.99)

e; very large change (ES ≥ 2.00); ES data indicate the differences compared with the pre values; CMJ, countermovement jump; Dm, muscle belly displacement; Tc, contraction time; DOMS, delayed onset muscle soreness; SRSS, short recovery and stress scale; OR, overall recovery; OS, overall stress; PAS, passive recovery; ACT, active recovery; CWI, cold water immersion; MAS, massage.

**Table 4:** Serum concentrations of creatine kinase, c-reactive protein, urea, free-testosterone and insulin-like growth factor 1 at 24 h before the half-marathon (pre), immediately after the recovery intervention (post_rec_) and 24 h after the half-marathon (post_24_).

Variable	Group	Pre			Post_rec_			Post_24_			Time	Intervention x time interaction
											*p*	*p*
CK (U/l)	PAS	254	±	183	376	±	214^c^	645	±	404^d^	0.001	0.091
	ACT	162	±	74	335	±	128^c^	861	±	665^e^		
	CWI	320	±	301	417	±	218^b^	615	±	220^c^		
	MAS	272	±	144	417	±	152^c^	655	±	291^d^		
CRP (mg/l)	PAS	1.43	±	1.56				5.28	±	3.65^d^	0.001	0.768
	ACT	0.90	±	1.03				3.87	±	2.04^d^		
	CWI	0.72	±	0.64				3.63	±	1.69^d^		
	MAS	0.43	±	0.25				2.58	±	1.07^d^		
Urea (mg/dl)	PAS	39	±	15				41	±	10^b^	0.001	0.883
	ACT	31	±	5				35	±	7^b^		
	CWI	35	±	10				40	±	9^b^		
	MAS	34	±	7				38	±	6^b^		
f-T (pg/ml)	PAS	14.2	±	5.0				12.4	±	4.5^b^	0.001	0.251
	ACT	15.0	±	4.2				9.7	±	3.8^d^		
	CWI	14.4	±	4.6				11.5	±	4.4^c^		
	MAS	17.5	±	10.0				13.1	±	7.5^c^		
IGF-1 (ng/ml)	PAS	242	±	72				223	±	75^b^	0.001	0.112
	ACT	229	±	69				212	±	67^b^		
	CWI	206	±	57				193	±	43^a^		
	MAS	277	±	90				230	±	79^b^		

Data are shown as the mean ± SD

a, trivial change (ES = 0.00–0.19)

b, small change (ES = 0.20–0.59)

c, moderate change (ES = 0.60–1.19)

d, large change (1.20–1.99)

e, very large change (ES ≥ 2.00); ES data indicate the differences compared with the pre values; CK, creatine kinase; CRP, c-reactive protein; f-T, free-testosterone; IGF-1, insulin-like growth factor 1; PAS, passive recovery; ACT, active recovery; CWI, cold water immersion; MAS, massage.

At post_rec_, ACT was harmful to perceived recovery (ACT vs. PAS: effect size [ES] = -1.81) and serum concentration of CK (ACT vs. PAS: ES = 0.42). CWI was harmful to CMJ performance (CWI vs. PAS: ES = −0.98) and beneficial for reducing muscle soreness (CWI vs. PAS: ES = −0.88) and perceived stress (CWI vs. PAS: ES = −0.64). MAS was beneficial for reducing muscle soreness (MAS vs. PAS: ES = −0.52) and improving perceived recovery (MAS vs. PAS: ES = 1.00).

At 24 h, ACT was harmful to perceived recovery (ACT vs. PAS: ES = −0.68) and to the serum concentrations of CK (ACT vs. PAS: ES = 0.84) and f-T (ACT vs. PAS: ES = −0.91). Both CWI and MAS were still beneficial for reducing muscle soreness (CWI vs. PAS: ES = −1.49; MAS vs. PAS: ES = −1.12), with MAS being harmful to Dm (MAS vs. PAS: ES = 0.42) and the serum concentration of IGF-1 (MAS vs. PAS: ES = −0.34).

In addition, all markers showed a significant fatigue-related change over time (p < 0.05) (Tables 3 and 4). A significant recovery intervention × time interaction was found for CMJ height, muscle soreness and perceived recovery (Figs 1 and 2).

## Discussion

The present investigation examined the effects of a single bout of either ACT, CWI or MAS on the fatigue markers in trained long-distance runners following a half-marathon run. The collected data indicate that the half-marathon race induced a temporary state of fatigue independent of the recovery mode, as significant fatigue-related alterations in muscle contractile characteristics, perceptions of muscle soreness, recovery and stress as well as in blood markers of muscle damage (i.e. CK), inflammation (i.e. CRP), metabolic status (i.e. urea and IGF-1) and neurohumoral regulation (i.e. f-T) occurred. This finding confirms the findings of other research studies that demonstrated a significant increase in the symptoms of fatigue after severe prolonged exercise [1, 3, 10–12, 31–34]. In relation to the comparison among the different recovery interventions, the major finding of this study is that CWI and MAS can be recommended for restoring subjective fatigue measures because these recovery strategies are more effective than PAS, with ACT being even disadvantageous. However, runners must be aware that neither the use of ACT nor CWI or MAS had any beneficial effect on objective fatigue markers, whereas ACT even had a negative impact in some cases.

Many athletes regularly perform short-duration (i.e. approximately 15 min), low-to-moderate-intensity exercises (referred to as ACT) within approximately 1 h after their practice or competition in an attempt to facilitate psychophysiological recovery and to restore exercise performance. However, the findings of this study do not support the use of a running-based active cool-down within 1 h after a severe prolonged endurance exercise to improve recovery. Compared with PAS, ACT had no acute (post_rec_) and/or medium-term (post_24_) effects on the loss of contractile capacity of the rectus femoris muscle and on muscle soreness, perceived stress and indirect markers of inflammation and metabolic status. ACT even had a negative effect on perceived recovery and on the structural muscle damage and neurohumoral regulation assessed by circulating CK and f-T. This result supports recently published reviews reporting that ACT is largely ineffective for improving most psychophysiological markers of post-exercise recovery [7, 35].

Conversely, some authors reported the beneficial effects of an active cool-down in contrast to the results of this study. For example, Gill, Beaven, & Cook [36] demonstrated an enhanced clearance rate of CK as a result of ACT performed immediately post-exercise. These inconsistent results on CK activity, together with the detrimental effect of ACT on perceived recovery and circulating f-T shown in this study, may be explained by the selection of the type of exercise in ACT. In the study by Gill et al. [36], ACT was performed on a cycle ergometer, whereas in the current investigation, the active cool-down involved slow running. In this respect, the use of running as a type of ACT is assumed to likely increase the time before muscle regeneration sets in and perhaps even aggravate the symptoms of fatigue as a result of sustained eccentric biomechanical strain on the working muscles [37]. Therefore, if ACT is used as a recovery strategy, activities such as cycling, rowing, or water-based activities (e.g. swimming, aqua biking, pool walking/jogging and water aerobics), in which the body weight is borne by an external element and the eccentric strain is consequently reduced, are probably better suited.

Besides ACT, the use of post-exercise CWI and MAS is gaining considerable popularity among athletes in many kinds of sports to accelerate recovery. The results of the present study show that compared with PAS, CWI negatively affects muscle performance at post_rec_. An acute impairment of muscular performance following CWI was previously reported [38, 39] and proposed to be related to the detrimental effect of cold on muscle temperature and nerve velocity conduction as well as to a parasympathetic reactivation by CWI [23, 40]. In this context, CWI is assumed to affect the exchange between Ca^2+^ and Na^2+^ in neural cells, possibly leading to a delay in action potential generation, contraction speed and force-generating capacity, which consequently reduces the dynamic contractile force and thus subsequent performance [23]. However, this effect can be neglected by runners because they usually do not have to complete subsequent events on the same day.

Moreover, CWI had a positive effect on muscle soreness at post_rec_ and post_24_ and on perceived stress at post_rec_ in comparison with PAS. By contrast, no acute (post_rec_) and/or medium-term (post_24_) effects on perceived recovery or on any of the objective fatigue markers were found for CWI. The positive (both acute and medium-term) effects of CWI on post-exercise muscle soreness are consistent with the majority of previous research on the topic, as reported in the reviews by Poppendieck et al. [8], Machado et al. [23], Ihsan et al. [40], Dupuy et al. [41] and Leeder et al. [42], and confirm that CWI appears to be an effective analgesic. However, the literature findings examining CWI as a recovery tool are rather inconclusive, with applied research showing also unchanged [43, 44] or even impaired [45, 46] recovery of fatigue markers following CWI. In this context, Machado et al. [23] and Ihsan et al. [40] highlighted that the efficacy of CWI for attenuating symptoms of fatigue (i.a. muscle soreness) seems dependent on the CWI protocol used and on the mode of exercise that causes the fatigue. For example, CWI application demonstrated limited recovery benefits when fatigue was induced by single-joint, unilateral movements. By contrast, CWI was more effective in ameliorating the effects of muscle damage induced by whole-body prolonged endurance or intermittent-based exercises [40]. Moreover, Machado et al. [23] provided evidence of the presence of a dose–response relationship with CWI, indicating that a water temperature of 11°C–15°C and an immersion time of 11–15 min could lead to the best results. Consequently, the present findings may not be applied to other athletic activities and/or if different CWI protocols are used.

As described above, the acute analgesic effect of CWI is, among other things, associated with the reduction of nerve conduction. In this context, cold exposure has been shown to activate the transient receptor potential cation channel M8 (TRPM8) receptors. Once activated, TRPM8 mediates analgesia through inhibitory inputs either through spinal inhibitory interneurons or directly to nociceptors [40], which in turn may modulate the sensation of acute muscle soreness and also improve the perception of stress found at post_rec_ after CWI. Interestingly, CWI still had an analgesic effect on muscle soreness 24 h after the half-marathon. Whether this is a true physiological effect or simply reflects a placebo effect remains unclear. However, given the perceptual nature of the muscle soreness assessment, it is plausible that the placebo effect phenomenon may affect the efficacy of CWI as a recovery strategy. Owing to the increasing popularity of CWI, the participants could have believed and expected a positive outcome from CWI, thereby influencing the measure of muscle soreness and the perception of stress [42]. This finding is also be supported by the fact that CWI had no effect on any of the objective fatigue markers. In addition, Broatch et al. [47] found that CWI was no more effective than a placebo immersion protocol at improving recovery. Thus, many of the hypothesised physiological benefits surrounding CWI were suggested to be at least partly placebo related and that treatment belief was a powerful element [10].

In this study, MAS post-race did not appear to alleviate the physiological symptoms of fatigue, such as the loss of contractile capacity of the rectus femoris muscle and changes in blood markers of fatigue, faster than PAS. At post_24_, there was even a small harmful effect of MAS in comparison with PAS on Dm and IGF-1. By contrast, MAS had a positive medium-term (post_24_) and/or acute (post_rec_) effect on muscle soreness and perceived recovery. Given the substantial inconsistencies in the research findings on MAS and its efficacy for recovery, these inconsistencies could be attributed to the large variety of massage techniques used, the individual skill of the therapist, the duration and timing of the MAS and the type of the fatigue-inducing exercise [9, 48]. The results of this study are both consistent and in contrast to those of previous studies. However, in their review articles, Poppendieck et al. [9], Nédélec et al. [48], Barnett [49], Bishop et al. [50] and Weerapong et al. [51] concluded that most of the evidence points towards MAS being effective in alleviating muscle soreness and improving perceptions of recovery and stress, but its effect on the physiological fatigue markers remains unclear. This finding is in agreement with the result of the present study and those of Dawson et al. [11] and Visconti et al. [52], who reported that MAS did not affect the physiological measures of fatigue but did affect perceived recovery and muscle soreness following a half-marathon and an ultramarathon.

Some studies explained the mechanism of muscle soreness reduction through MAS by the increase in neutrophils and the reduction of serum concentration of CK, whereas some researchers failed to explain the mechanism at all [51]. As only muscle soreness sensation (i.e. the subjective measure reported by the runners) was reduced after the MAS application in this study, the MAS treatment could have provided only a psychological advantage with no clear benefits of MAS on physiological fatigue. Moreover, the improvement in lowering perceived pain through MAS could also have a positive effect on perceived recovery. A reduction in the circulating cortisol and an increase in the concentration of beta-endorphins were also proposed to explain the reduction in perceived fatigue following MAS [41]. The precise reason for the small negative effect of MAS on Dm and IGF-1 found in this study remains unclear, as MAS did not affect any of the other objective fatigue markers. However, note that the MAS of injured tissue may lead to further muscle damage if given immediately after an exercise session that induces muscle damage [48, 49], which in turn may aggravate some symptoms of fatigue. In addition, the change in Dm may also be random, since the CV for Dm is 12.9%, which is greater than the observed difference in the change of Dm between MAS and PAS.

The potential limitations of the study design should also be addressed. First, because of limited time and resources, we could not use a crossover design to investigate the effect of the three different recovery strategies on the fatigue markers following a half-marathon (i.e. the runners would have had to complete a total of four half-marathons in a crossover design), but this design could have had some advantages over a parallel design. For example, we would have been able to compare and report intraindividual responses to the treatments (i.e. ACT, CWI, MAS and PAS). Second, again because of limited time and resources, we could only collect data at pre, post_rec_ and post_24_. However, a larger number of measurement times would have helped to better reflect the time course of recovery. Third, we did not perform a sample size estimation. However, compared with similar studies, the number of participants was relatively high in the present study. Fourth, given the practical difficulty of blinding the participants, investigators and outcome assessors from the recovery strategies, the potential beneficial effect arising from the placebo effect of the recovery techniques could not be eliminated. The fact that the implementation of MAS or CWI after the half-marathon resulted only in positive effects on the subjective measures of fatigue lends further weight to the suggestion that any effects attributed to these recovery modalities could be a product of the placebo effect. Therefore, future research should be conducted to implement effective placebo interventions in place of a control group (although this is difficult or even impossible in research on recovery strategies) or to at least consider a measure of treatment belief when comparing different recovery interventions [10].

## Practical applications

Recreational runners should be aware that a half-marathon race leads to substantial changes in physical and subjective measures of fatigue. Therefore, after such a competitive race, adequate recovery periods should be planned. As a running-based active cool-down even has a negative effect on recovery, runners are advised to focus on other recovery modalities to minimise the severity of fatigue rather than to run at low intensities. Conversely, the use of CWI and/or MAS after a severe prolonged endurance exercise may be recommended with limitations. Both CWI and MAS have beneficial effects on the subjective measures of fatigue (i.e. muscle soreness and perceived recovery/stress) and may increase well-being and the perception of better preparedness for subsequent exercise. This feel-better phenomenon may be critical for the restoration of exercise performance because performance has been shown to be impaired in the presence of muscle pain regardless of the extent of exercise-induced muscle damage. This finding is in accordance with the growing body of evidence implicating central nervous system-mediated mechanisms in facilitating athletic recovery [40, 53].

However, perceptual recovery is not necessarily synchronised with the degree of the loss of exercise capacity or the indicators of immune changes and muscle damage even without the application of any recovery technique. For example, Stacey et al. [54] showed that the use of CWI did induce greater perturbation to blood immune markers than the use of PAS, and that it improved perceptions of recovery even though the subsequent performance was not affected. Consequently, despite the enhanced perceptual recovery (e.g. due to the application of a recovery method), there may still be (half-)marathon-induced immune perturbations (also referred to as the ‘open window’ of immune dysfunction), during which viruses and bacteria gain foothold and the risk of subclinical and clinical infections (e.g. upper respiratory tract infections such as the common cold) is increased [55]. Additionally, if CWI or MAS promotes perceptual recovery without an accompanying physiological and/or performance recovery, the runner may attempt subsequent exercise loads beyond the runner’s current capacity. This action may lead to inappropriate levels of stress, undesirable levels of fatigue and a potentially greater risk of injury [49].

Furthermore, as long as a recovery strategy has no negative side effects, individual preferences as well as experiences and beliefs may influence the choice of whether these methods are applied post-exercise. For example, there is a strong preference for using CWI after long-distance running in the heat, and CWI is probably less popular than the other strategies to be performed after exercise in a cold, wet and/or windy environment. Whether the organisers provide the possibility of applying CWI and/or MAS after a race should also be observed. However, in this context during running events, the hygiene standards and the necessary water temperature in connection with CWI are difficult to meet, and MAS often requires a long wait. Therefore, runners may need to arrange themselves in a certain way to avail of CWI or MAS, for example, in a nearby hotel or swimming pool with a wellness area.

Finally, it has to be taken into account that the current investigation was based on the effects of a single use of either ACT, CWI or MAS. However, a one-time application of a recovery modality after strenuous exercise may not be sufficient to effectively promote recovery. Therefore, further research is warranted to investigate the effect of a repeated and/or prolonged use of ACT, CWI or MAS following long-duration running exercises.

## Conclusions

For recreational runners, a half-marathon race can result in fatigue symptoms lasting at least 24 h. To restore subjective fatigue measures, CWI and MAS can be recommended because these recovery strategies are more effective than PAS, with ACT being even disadvantageous. However, runners must be aware that neither the use of ACT nor CWI or MAS had any beneficial effect on objective fatigue markers.

## Supporting information

S1 File Study data. Rec, recovery; CMJ, countermovement jump; Dm, muscle belly displacement; Tc, contraction time; DOMS, delayed onset muscle soreness; SRSS, short recovery and stress scale; OR, overall recovery; OS, overall stress; CK, creatine kinase; CRP, c-reactive protein; f-T, free-testosterone; IGF-1, insulin-like growth factor 1.
(XLSX)
